# Methods for interpreting lists of affected genes obtained in a DNA microarray experiment

**DOI:** 10.1186/1753-6561-3-s4-s5

**Published:** 2009-07-16

**Authors:** Jakob Hedegaard, Cristina Arce, Silvio Bicciato, Agnès Bonnet, Bart Buitenhuis, Melania Collado-Romero, Lene N Conley, Magali SanCristobal, Francesco Ferrari, Juan J Garrido, Martien AM Groenen, Henrik Hornshøj, Ina Hulsegge, Li Jiang, Ángeles Jiménez-Marín, Arun Kommadath, Sandrine Lagarrigue, Jack AM Leunissen, Laurence Liaubet, Pieter BT Neerincx, Haisheng Nie, Jan van der Poel, Dennis Prickett, María Ramirez-Boo, Johanna MJ Rebel, Christèle Robert-Granié, Axel Skarman, Mari A Smits, Peter Sørensen, Gwenola Tosser-Klopp, Michael Watson

## Abstract

**Background:**

The aim of this paper was to describe and compare the methods used and the results obtained by the participants in a joint EADGENE (European Animal Disease Genomic Network of Excellence) and SABRE (Cutting Edge Genomics for **S**ustainable **A**nimal **Bre**eding) workshop focusing on post analysis of microarray data. The participating groups were provided with identical lists of microarray probes, including test statistics for three different contrasts, and the normalised log-ratios for each array, to be used as the starting point for interpreting the affected probes. The data originated from a microarray experiment conducted to study the host reactions in broilers occurring shortly after a secondary challenge with either a homologous or heterologous species of *Eimeria*.

**Results:**

Several conceptually different analytical approaches, using both commercial and public available software, were applied by the participating groups. The following tools were used: Ingenuity Pathway Analysis, MAPPFinder, LIMMA, GOstats, GOEAST, GOTM, Globaltest, TopGO, ArrayUnlock, Pathway Studio, GIST and AnnotationDbi. The main focus of the approaches was to utilise the relation between probes/genes and their gene ontology and pathways to interpret the affected probes/genes. The lack of a well-annotated chicken genome did though limit the possibilities to fully explore the tools. The main results from these analyses showed that the biological interpretation is highly dependent on the statistical method used but that some common biological conclusions could be reached.

**Conclusion:**

It is highly recommended to test different analytical methods on the same data set and compare the results to obtain a reliable biological interpretation of the affected genes in a DNA microarray experiment.

## Background

The previous Microarray Data Analysis Workshop organised by EADGENE (European Animal Disease Genomic Network of Excellence, [1]) in November 2006, focussed on the analytical methods applied to raw microarray data to obtain lists of significantly affected genes. The results from the workshop were published in Genetics Selection Evolution [2-5]. This paper summarises the results obtained from a joint EADGENE and SABRE (Cutting Edge Genomics for **S**ustainable **A**nimal **Bre**eding, [6]) workshop in November 2008, focusing on the interpretation of lists of significantly affected genes, thereby extending the work from the previous workshop. The aim of the workshop was to evaluate and present existing methods and softwares, and potentially to propose new methods to deal with the post-analyses of microarray data, using real data sourced from within EADGENE and SABRE.

The initial objective of an analysis of a microarray data set is to produce a list of significantly affected probes/genes. This analysis can be relatively challenging, but the major challenge is to interpret the list of hundreds to thousand affected genes and draw some biological conclusions. To assist this process, a large number of statistical methods using quite different approaches have been proposed, which can consequently produce different results if applied to the same data set [7,8]. Gene-set analysis is a popular method and aims to identify differentially expressed gene sets associated with e.g. a phenotype of interest. Gene sets are commonly defined based on existing biological knowledge on gene function available from public databases, such as Gene Ontology (GO) [9,10], Kyoto Encyclopaedia of Genes and Genomes (KEGG) [11,12] and Gene Map Annotator and Pathway Profiler (GenMAPP) [13,14]. Current available tools for gene-set analysis have recently been reviewed by Huang *et al *[15] who define three classes of tools according to their underlying algorithms: singular enrichment analysis; gene set enrichment analysis; and modular enrichment analysis. Singular enrichment analysis (SEA) is a widely used approach, which utilises gene sets derived from Gene Ontology or pathway databases and investigates the enrichment of specific gene sets in a list of significantly affected genes, defined by applying a cut-off threshold value. SEA suffers from the use of a cut-off threshold value, the level of which has a major impact on the obtained results [16]. To avoid this problem, a group of methods termed gene set enrichment analysis (GSEA) have been developed, which utilise the information from all probes/genes in a microarray experiment. Modular enrichment analysis (MEA) is based on SEA but integrates term-term/gene-gene relationship to reveal biological meaning not revealed by single term/gene analysis.

A common challenge faced during the interpretation of the affected probes is the lack of appropriate annotation of the probes on the microarray. An affected probe without annotation can consequently not contribute to the interpretation of the results and if a major fraction of the probes are without annotation it may have a negative influence on the following analysis, such as GO enrichment analysis. A study of methods to improve the annotation of microarray probes was also a part of this workshop and is described in the adjacent papers [17-20].

In this paper, the methods applied and the results obtained by the participating groups are summarised and some general conclusions are drawn.

## Methods

### The data – host reactions in broilers after a secondary challenge

*Eimeria *are obligate intracellular protozoan parasites which can affect chickens and continuous exposure to *Eimeria *can result in protective immunity. The process leading to protective immunity was investigated by studying the host reactions after homologous or heterologous secondary infections. A total of 125 one-day-old Ross 308 male broilers were randomly divided in five groups of 25 broilers each. At 7 days of age, three groups were inoculated with phosphate buffered saline (P) and two groups were inoculated with *E. maxima *(M). A secondary challenge followed at day 21 of age. This challenge was with PBS (P), *E. maxima *(M) or with *E. acervulina *(A), forming five challenge groups PP, PM, PA, MM and MA. Five chickens from each group were killed at 8 and 24 hours after the second challenge and specific regulations of gene expression profiles in the jejunum were monitored using chicken whole genome oligonucleotide microarrays (ARK-Genomics *Gallus gallus *20 K v1.0). The obtained microarray data was normalised and analysed and lists of affected genes were obtained for different contrasts. The result of the contrasts MM8-PM8, MM8-MA8 and MM8-MM24 were provided for this workshop as three lists including all microarray probes and test statistics for the three different contrasts. The number of affected probes for each contrast is shown in Table 1. The normalised log-ratios for each array were furthermore provided to the participating teams. The contrasts address different biological questions: differences between secondary and primary challenge (MM8-PM8), differences between homologous and heterologous challenge (MM8-MA8) and differences between two time points of a homologous challenge (MM8-MM24). The microarray data is available at the ArrayExpress database [21] under accession number E-MEXP-1972 and the three gene lists can be found as supplementary material to this paper (See Additional file 1). The data set used in this paper is part of larger data set, which includes additional time points, and a paper describing this complete set is in preparation by Rebel *et al*.

**Table 1:** The contrasts used in the workshop. The number of significantly (FDR <= 0.05) affected probes for the three different contrasts used in the workshop.

Contrast:	MM8.PM8	MM8.MA8	MM8.MM24
Repressed	803	58	639
Induced	923	23	152

### Annotation of the chicken microarray probes

A proper annotation of the individual probes on a microarray is a prerequisite for establishing a link between the probe and the associated biological knowledge such as gene ontology and pathways. The annotation files used for interpreting the three provided gene lists were the ones obtained as a part of the workshop and described in the adjacent papers [17-20]. The most recent versions of the annotation files are available at the EADGENE Oligo Set Annotation Files homepage [22]. Version 2, released September 11^th ^2008, based on Ensembl version 50 was used for the workshop. Furthermore, the group from the Wageningen University built a customized annotation utilising chicken-human orthologous gene information and performed separate analyses for each annotation [23], the group from Aarhus University investigated methods for predicting the possible annotations for genes with unknown function from the expression data [24] and the participants from Institute for Animal Health based their analysis on an annotation obtained with the IMAD system (see [18] for additional details).

### Analysis of the data

The participating groups applied conceptually different analytical approaches to interpret the three provided gene lists, using both commercial and public available software (Table 2). The main focus of the approaches was to utilise the relation between probes/genes and their gene ontology and pathways to interpret the affected probes/genes. The issue of correction for multiple testing was discussed by several groups. Additionally, as 2420 ENSEMBL chicken genes were found to be represented by multiple (up to nine) oligonucleotides on the chicken microarray, the effects of using the data from individual oligonucleotides or from the genes (represented by multiple oligonucleotides) were studied by the group from Aarhus University [24].

**Table 2:** Summary of the analytical approaches and choice of software for each group

**Group**	**Analytical tool**	**Class^a^**	**Key statistical method**
University of Cordoba, Spain [25]	ArrayUnlock [31]	MEA	Hypergeometric; Chi Square-test
	
	Ingenuity Pathway Analysis [32]	SEA	Fisher's exact test

Institute for Animal Health, Compton, UK [27]	MAPPFinder/GenMAPP [14,33,34]	SEA	Z-score; Hypergeometric

Aarhus University, Denmark [24]	LIMMA [35]	SEA	Wilcoxon
	
	TopGO [30]	MEA	Fisher's exact test; Kolmogorov Smirnoff
	
	Globaltest [36]	GSEA	Global test
	
	GIST [37]	-	support vector machine classification; kernel principal components analysis

Animal Breeding and Genomics Centre, Wageningen University, Wageningen, The Netherlands [23]	AnnotationDbi [38]	-	-
	
	GOstats [39]	SEA	Hypergeometric

Animal Breeding and Genomics Centre, Animal Sciences Group, Lelystad, The Netherlands [28]	Globaltest [36]	GSEA	Global test
	
	GOEAST [40]	SEA	Fisher's exact; Hypergeometric; Chi Square-test

INRA, Toulouse and Rennes [26]	GOTM [41]	SEA, GSEA	Hypergeometric
	
	Pathway Studio [42]	-	Subnetwork Enrichment Analysis (SNEA), one-sided Mann-Whitney U-Test
	
	Ingenuity Pathway Analysis [32]	SEA	Fisher's exact test

^a ^SEA, singular enrichment analysis; GSEA, gene set enrichment analysis; MEA, modular enrichment analysis, as suggested by Huang *et al *[15]

## Results and discussion

### Annotation of the chicken microarray probes

The challenge of mapping the probes/genes on the chicken microarray to biological knowledge, such as gene ontology and pathways, was encountered by all groups. In general, half of the probes could be mapped and contribute to the biologically interpretation of the data. The lack of a well-annotated microarray did consequently have a detrimental effect on the results. Improvements were however obtained by using chicken-human orthologous gene annotation in contrast to chicken gene annotation as reported by the group from Wageningen University [23]. The chicken-human orthologous gene information resulted in a higher power to detect significant GO terms due to the higher coverage of GO terms assigned to human genes comparing to chicken genes, but as human and chicken are evolutionarily rather far apart care has to be taken when interpreting the obtained results [23]. The group from Aarhus University investigated methods for predicting the annotations of genes with unknown function from the expression data, and found that the methods may be of potential use, but that improvements in the chicken annotation, availability of larger microarray data sets and careful validation of the predictions are needed to fully utilise these methods [24].

### Analysis of the data

The results of the different analytical approaches applied by the participating groups showed, in general, that the biological interpretation is highly dependent on the statistical method used.

The analysis for enrichment of GO-terms based on singular enrichment analysis (SEA), applied by the different groups (Table 2), revealed differences in numbers and identity of the GO-terms found to be affected. In general, many of the enriched GO-terms were found to be represented by few (1 or 2) genes. Applying the commonly used filtering criteria, requiring a reasonable number of genes, e.g. 10, to represent each GO-term, would lead to the conclusion that very few GO-terms are affected.

The commercial software Ingenuity Pathway Analysis (IPA) was used by the groups from University of Cordoba, Spain [25] and from INRA, Toulouse and Rennes [26] to explore the affected pathways. The results obtained by the two groups are quite similar even though the analyses were performed in different ways. In contrast to the group from University of Cordoba, the INRA group compared the networks of the three gene lists to the networks obtained from the complete list of genes on the microarray to identify significant networks relative to the microarray background.

Using GenMAPP/MAPPFinder, Prickett and Watson identified several biologically relevant pathways being affected, thus demonstrating the usefulness of this tool for microarray analysis, especially with an improved annotation [27].

The analytical methods based on gene set enrichment analysis (GSEA) (GlobalTest applied by the groups from ASG [28] and DJF [24]) did in general result in a larger number of terms to be significant than found using the SEA based methods. This was expected as theoretical considerations indicate that this method is more powerful [29].

The tool topGO belongs to the modular enrichment analysis (MEA) class of methods and takes the GO structure into account when testing the gene sets. Comparing the results obtained using topGO with the results from "classical" Fisher's exact test and the Kolmogorov Smirnoff test, both of which ignore the GO structure, showed that fewer significant terms were found with topGO [24], which may indicate increased specificity [30].

The majority of the analytical tools provide options for correction for multiple testing using various methods. It is common practice to apply multiple test correction to control the family-wise false-positive rate in the result list, but there is little consensus on how to perform the correction and whether the correction improves the results [15]. Several groups applied some methods for correction for multiple testing during their analysis of the data for this workshop, and found only a few significant terms/pathways after correction [24,27,28]. The essential problems are that the structure of the GO graph and pathways are in conflict with the assumption of independence and that most methods for multiple test correction do not change the ranks and therefore the relative importance of the different GO terms [24].

Where genes are represented by one or more oligonucleotides, it is possible to carry out enrichment tests at the level of the gene or at the oligonucleotide. These two levels could potentially produce different results. However only minor differences were found between enrichment tests at the level of the gene compared to those at the level of the oligonucleotide [24]. It is difficult however to generalize this result to other datasets but if the number of replicate probes varies for different genes it will often be better to use gene-based tests.

Despite differences in the specific GO-terms and pathways found to be affected by the groups, some common biological conclusions could though be reached for the three contrasts. Specific details of the biological conclusion can found in the papers from the participating groups [23-28]. The interpretation of the genes affected between MM8 and PM8 shows, as expected, that a secondary immune response is induced by the homologous challenge while the heterologous challenge induces a primary immune response. The lowest number of affected genes was found when comparing the expression profiles from homologous and heterologous challenge (MM8-MA8, table 1). This indicates that an *E. acervulina *infection triggers a similar response as an *E. maxima *infection. The identity of the affected genes between different time points of a homologous challenge (MM8-MM24) indicates that the secondary immune response increases from 8 to 24 hours.

## Conclusion

Different analytical methods were applied by the teams of the joint EADGENE and SABRE workshop focusing on the extraction of biological meaning from lists of significantly affected genes. The analyses were in general negatively affected by the lack of a well annotated microarray. However, the use of chicken-human orthologous gene annotation was found to improve the analyses. The results showed that the biological interpretation is highly dependent on the statistical method used but that some common biological conclusions could be reached. It is hence recommended to test different analytical methods on the same data set and compare the results to obtain a reliable biological interpretation of the affected genes in a DNA microarray experiment.

## Competing interests

The authors declare that they have no competing interests.

## Authors' contributions

JH drafted the manuscript and all authors read and approved the final version of the manuscript.

## Supplementary Material

Additional file 1 Microsoft Excel spreadsheet file including the output from the statistical tests of the three contrasts MM8-PM8, MM8-MA8 and MM8-MM24 on separate worksheets. Each worksheet contains 13158 rows and the following columns: Block, Row and Column: block number and row-column number in block; Gene.Name: 384 well source plate number and well and reporter name from the annotation version 1.0 for ARK-Genomics G.gallus Oligo Probe set; Name.descr: reporter ID from the annotation version 1.0 for ARK-Genomics G.gallus Oligo Probe set; logFC: estimate of the log2-fold-change corresponding to the effect or contrast; AveExpr: average log2-expression for the probe over all arrays and channels; t: moderated t-statistic; P.Value: raw p-value; adj.P.Value: p-value adjusted for multiple testing (FDR); B: log odds that the gene is differentially expressed. Click here for file
